# Supplementary material for: Expectation generation and its effect on subsequent pain and visual perception
Source: PLoS Comput Biol. 2025 May 22;21(5):e1013053. doi: 10.1371/journal.pcbi.1013053 (PMC12136418; doi:10.1371/journal.pcbi.1013053)
Supplement: Attachment — Submitted filename: BotvinikNezer_PPRI_PlosCompBiol_R2R.pdf [file pcbi.1013053.s003.pdf]

Response to Reviewers

We appreciate the insightful and thorough feedback from the reviewers, and the opportunity to respond. We address each comment below.

Reviewer 1

In this manuscript, participants (N = 45) underwent a vicarious learning paradigm where they were presented with fictitious ratings prior to stimulus delivery to explore the effects of variability, skewness, and mean expectation on pain and visual perception. The authors found results generally aligned with contemporary Bayesian perception models, although some interesting deviations were found. These effects were mostly driven by the visual domain rather than the pain domain. The study presents an interesting approach, but it cannot be recommended for publication in its current form due to a number of major and minor issues (see below).

Major Issues:

Comment 1.1:

1. The study design remains unclear in several aspects, such as the mention of "unreinforced trials" and the specifics of stimulus intensity levels and their relationship to the cue mean and participants' pain ratings. As I understand it, their study fundamentally differs from Yoshida and our replication, where the mean of the vicarious pain ratings was directly related to the subjects' perception of the given stimulus. Here, however, it seems the authors used fixed cue mean values (30 and 70), ignorant of what the actual presented intensity was and how it should be perceived based on the individual psychometric curve. This could undermine the validity of the cues if they are too far removed from the participant's actual perception, making them less credible. Additionally, details such as how many stimuli were presented and how often expectations were higher or lower only become partly clear at the end of the manuscript. I recommend that the authors revise the manuscript such that all relevant methodological information is presented before the results section.

Response 1.1:

We thank the reviewer for emphasizing that our description of the experiment design was not clear enough, and the importance of explaining the rationale and implications of using fixed, non-reinforced cues. First, we have now moved some of the methods descriptions from the Methods section to the relevant results subsection, for example:

Results

"In the cued-perception task (for a detailed description see Methods and Figure 1), participants viewed cues consisting of a distribution of 10 values (with a fixed mean value of 30 or 70, low or high variance,

and negative, symmetric or positive skewness) followed by painful heat (47°C or 48°C) or flickering checkerboards (50% or 60% luminance contrast). This task included 144 trials overall (three repetitions of each combination of modality, stimulus intensity, cue mean, cue variance and cue skewness)." (page 12).

This clarifies that each cue was based on a distinct, cue-specific distribution that varied in mean, variance, and skewness, though the mean value was indeed centered on 30 or 70. Second, we now explicitly state that the cues we have used were similar to several previous papers (e.g., Koban et al. 2016, 2018, 2019) but different from the cues used in Yoshida et al. and your replication, since the cues did not depend on the individual participant's psychometric curve. We explain our rationale as follows:

Introduction
"*Here, we test how expectations are generated from distribution cues with varying levels of mean, variance, and skewness (and thus the presence and direction of outliers), and how they affect subsequent perception of visual and painful stimuli (for the study design and illustration of the cues see Figure 1 and Methods). The cues were not externally reinforced (i.e., not predictive of subsequent stimulus intensity), and thus were informational but not subject to associative learning (i.e., they were not classically conditioned). They also did not depend on the participant's individually calibrated intensities, similar to several previous studies [8,10,64,65] (we note that in some other studies, cues were based on the individual's psychometric curve [7,9,37]). Such cues allow us to focus on effects of cognitive predictive information, rather than classical conditioning, which can occur when cues are associated with different levels of subsequent stimulus intensity and are thus truly informative about future intensity.*" (page 6).

Discussion
"Several limitations should be considered in the context of the current findings. First, we have used cues that were not predictive of actual stimulus intensity (i.e., non-reinforced), with fixed mean levels that did not depend on participants' individual psychometric curves (i.e., how intense the stimulus is expected to be perceived by the specific individual), and fixed stimulus intensity levels (that were not calibrated to each participant). The use of non-reinforced cues was by design, as this allowed us to study the effects of conceptual information on brain and behavior, without influences of conditioning of nociceptive responses or other forms of associative learning. Though it is perhaps surprising that such "mere suggestions" without true information about the stimulus can have strong effects, such cues have been shown to yield robust behavioral effects that are comparable in size to those of cues that are predictive of actual intensity [10,64,65]. We also found similarly robust effects here, which decreased but persisted over the course of the experiment. The persistence of the effects over time here is also perhaps surprising based on the notion that even conditioned responses will generally extinguish if cues do not predict unconditioned stimuli in a valid way. However, this persistence has been replicated in multiple studies [10,64,66], and may be explained by a 'self-reinforcing' feedback loop detailed in Jepma et al. 2018 [66], whereby initial expectations influence both stimulus processing and learning to create persistent effects of initial beliefs." (page 28).

Finally, the behavioral effects and their durability in our study and previous ones with similar cues provide evidence that the cues are valid. The suggestion that we might observe a weakened cue effect with large discrepancy between cue value and stimulus intensity is an interesting one, and indeed one that we have

considered in past work (and have generally not found) and now consider more carefully here at the Reviewer's suggestion. If people notice a large discrepancy between the cue and stimulus and discount the cue as non-informative, this would produce a 'boundary effect', in which perception is less affected by cues that are too far from the actual incoming information, as has been previously demonstrated by Hird et al. [1] in pain. This effect is in distinction from 'assimilation effects', a movement towards the cue value, signaled by a linear effect of the cue-based prediction error. To address the potential for boundary effects, we performed an additional analysis similar to the one performed by Hird et al. to test for such boundary effects. For each trial, we defined the cue PE as the difference between the subjective stimulus value (defined as the participant's average rating of this stimulus intensity in the stimulus-response task) and the cue mean (this was called PE in Hird et al.), and experienced PE (PE_sub in Hird et al.) as the difference between the reported rating and the subjective stimulus value. Thus, the cue PE reflects the difference between cue-based expectations and sensory input, and experienced PE reflects the difference between the sensory input and the reported pain. We used the following mixed-effects model, per modality, to test for boundary effects:

$$experienced\_PE \sim cue\_PE + cue\_PE^2 + cue\_PE^3 + (1 \mid participant)$$

This model is referred to as the "simple model" in Hird et al.
We also ran Hird et al.'s "complex model", which adds *cue_PE*^4, the trial number and the interaction between the different degrees of *cue_PE* and the trial number:

$$experienced\_PE \sim 1 + cue\_PE + cue\_PE^2 + cue\_PE^3 + cue\_PE^4 + trial\_number + cue\_PE*trial\_number + cue\_PE^2*trial\_number + cue\_PE^3*trial\_number + cue\_PE^4*trial\_number + (1 \mid participant)$$

(note that adding *cue_PE* as a random slope caused the model to not converge, and thus this term was removed).

In both models, and for both modalities, the effect of the *cue_PE* was significantly negative, indicating that pain assimilates towards the cued values ($p < .001$). However, we did not find significant cubic (*cue_PE*^3) or quadric (*cue_PE*^4) effects, including no interactions of *cue_PE*^3 or *cue_PE*^4 with trial number, consistent with a lack of boundary effects for both modalities.
We also plotted *experienced_PE* as a function of *cue_PE* (as in Figure 3 by Hird et al., 2019). In pain, there seems to be some tendency towards boundary effects, but it was not strong enough to reach significance. In contrast, in the visual modality, the effect of the cue seems to (descriptively) increase, rather than decrease, as the *cue_PE* increases (*cue_PE*^3: pain $p = 0.102$; vision $p = .243$).

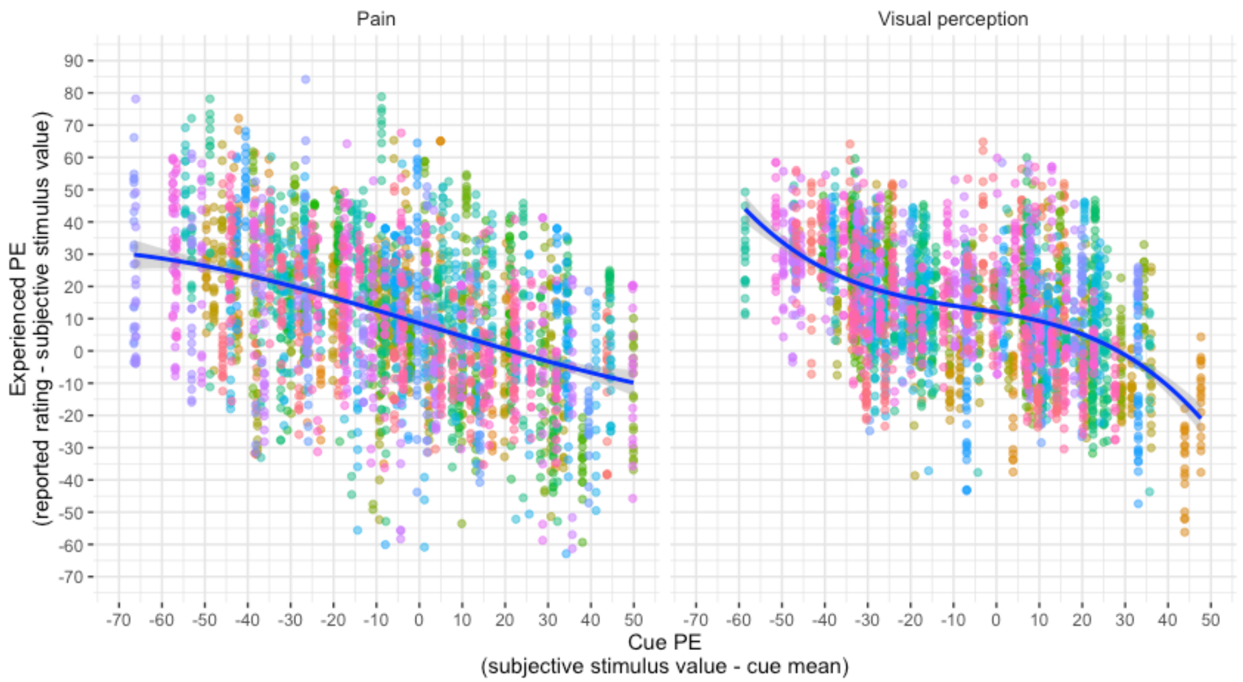

Figure S1. Boundary effects on cue-based expectations. The plots show *experienced_PE* by *cue_PE* for each modality. Different colors represent different participants. A third degree polynomial is shown on top of single-trial points (the gray shade represents the standard error).

We have now added this analysis to the main text, and new Figure S1 to the paper:

Methods

"Boundary effects on cue-based expectations were tested with mixed-effects models replicating Hird et al. [43]. The subjective stimulus value was computed as the averaged ratings of stimuli with the same intensity in the stimulus-response task, for each participant and modality. The cue PE (PE in [43]) was computed as the difference between the subjective stimulus value (sensory input) and the cue mean. Experienced PE (PE_sub in [43]) was computed as the difference between the reported rating and the subjective stimulus value. The "simple model" included experienced PE as the dependent variable (numeric), and cue PE, (cue PE)^2 and (cue PE)^3 (all numeric, non-centered) as predictors. The "complex" model included additional regressors and interactions: (cue PE)^4, trial number (per modality), and the interaction between each level of cue PE (cue PE, (cue PE)^2, (cue PE)^3, (cue PE)^4) and the trial number. Note that only a random intercept was included, since the model did not converge with random slopes. We report the results based on the complex model, but similar patterns were revealed with the basic model." (page 35).

Results

*"**"Boundary effects" of cue-based expectations.** Previous studies have demonstrated that the effect of cue-based expectations on pain perception decreases when the difference between the sensory input and the expectation is too large [43]. In the current study, we have used fixed stimulus intensities and cue mean values (i.e., these values were not calibrated to each participant), which could potentially result in large discrepancies for some participants, inducing such boundary effects. To test for such effects, we performed additional analyses similar to those of Hird et al. [43], testing cubic and quadratic effects of the cue PE*

(the difference between the subjective stimulus value, based on the average rating per participant, modality and intensity from the stimulus-response task, and the cue mean) on the experienced PE (the difference between the reported rating and the subjective stimulus value; for additional details see Methods). In both modalities, the model revealed a significant effect of the cue PE on experienced PE (pain: b = -0.460, SE = 0.056 , t(2955) = -8.142, p < .001; visual perception: b = -0.282, SE = 0.043 , t(2981) = -6.574, p < .001), replicating our finding of a significant assimilation of perceptual ratings towards the cue mean value. However, there were no significant cubic or quadratic effects in both modalities (pain: cubic t(2958) = 1.120, p = .263, quadratic t(2961) = 1.032, p = .302; visual perception: cubic t(2996) = 0.157, p = .876, quadratic t(2996) = -1.225, p = .221), providing evidence against the presence of boundary effects in our data (Figure S1)." (page 16).

Discussion

"Predictive processing theories also emphasize coding of PEs ('predictive coding' [6,68,90]), a contrast with predictions that provide efficient representations of salient changes in the environment and drive the updating of internal models (learning) [32,82,91]. A previous study has demonstrated "boundary effects" on cue-based expectations, such that the effect of cues on perception was decreased when the actual sensory input deviates too much from expectations [43]. We did not find evidence for such boundary effects in our data, although notably we did not systematically vary the PE, and the subjective stimulus values used to compute the PE were based on ratings provided in the stimulus-response task, which was performed on a different day from the cued-perception task." (page 25).

Comment 1.2:

The analysis of both modalities at once is confusing, especially given that the authors later on write that the pain and vision ratings are not directly comparable (making one questioning on the validity of these combined analysis then). Furthermore, while the interaction between modalities is often insignificant, the authors proceed to test them separately as if an interaction was present. This leads to considerable confusion, as many subsequent inferences about the implications for the pain field are based on general patterns across both modalities, even though these patterns do not hold when examining the pain data alone. I would propose focusing on the analysis separately per stimulus modality.

Response 1.2

We originally planned to report each modality separately, and use a combined model to report interactions with modality and generalizable effects. However, we agree that the analyses including models with both modalities and an interaction term in addition to separate models per modality (even in the absence of a significant interaction) are confusing. In accordance with the reviewer's suggestion, we now focus first on analyses of each modality separately, and have now revised the manuscript to focus on the separate models. Since all numerical variables are z scored, the estimates of each model are standardized and thus comparable across models, which allows us the ability to compare effect sizes across modalities. We have now revised the text accordingly, for example:

Results - expectation generation from distribution cues

"Expectation ratings significantly assimilated to the cue mean in both modalities (linear mixed effects model, pain: β = 0.901, SE = 0.015 , t(57.7) = 62.72, p < .001; vision: β = 0.9135, SE = 0.021 , t(51.3) = 43.25, p < .001)." (page 8)

Comment 1.3

I struggled to interpret several of the results due to unclear descriptions of the models, all tested predictors, etc. In addition, in Fig. 4C, there are very strong Block (due to repositioning) and Trial effects (habituation) that remain unclear whether they are always accounted for in the analysis and whether results remain preserved if included. Furthermore, the computational models discussed at the end of several results sections add to the confusion, as they are only explained in the supplementary materials, and the conclusions drawn from individual-level model preferences or group-level model comparisons are not always in line with the conclusions in the context where the authors refer to them.

Response 1.3

We thank the reviewer for pointing to these required clarifications. All variables included in each model are described in the Methods section. We now also describe them in the results section. We did not control for block (run) and trial in the previous manuscript version, but we agree with the reviewer that a more complete model is preferred, and in the revised manuscript we have added these regressors to all models. Note, however, that in the stimulus-response task there was only one block per modality, and thus we only added the trial number.

Results

"In the first task, participants rated the painfulness of thermal stimuli and the visual contrast of flickering checkerboards with five levels of intensities per modality (45°C, 46°C, 47°C, 48°C and 49°C, and 5%, 27.5%, 50%, 72.5%, and 95% luminance contrast; for more details see Methods and Figure 1), and 50 trials overall (5 trials per intensity level of each contrast). We tested the effect of the stimulus intensity on the rating with a separate mixed-effects model per modality, controlling for the trial number. The trial number covariate modeled variation in outcomes (pain or visual intensity) over time As expected, participant ratings were higher for higher stimulus intensity levels in both modalities (linear mixed-effects model: pain: β = 0.633, SE = 0.028, $t_{(44)}$ = 22.42, p < .001; vision: β = 0.867, SE = 0.018, $t_{(44)}$ = 47.06, p < .001)." (page 7)

*"**Expectation generation from distribution cues***

…

The effects of cue mean, variance, and skewness on reported ratings were tested simultaneously with mixed effects models (one per modality) including predictors for each distributional property and their interactions, and controlling for block and trial number within each block." (page 8)

"***The effects of cue-based expectations on perception***

…

The effects of stimulus intensity and cue mean, variance, and skewness on reported ratings were tested simultaneously with mixed effects models (one per modality) including these predictors and their interactions, as well as block number and trial number within block as additional numeric regressors (to control for temporal effects such as habituation and sensitization [72])." (page 12)

Controlling for trial and block did not change the results of the stimulus-response task and the expectation tasks, and the vast majority of the findings we report were unaffected by the additional covariates. However, the results of the cued-perception task did change in one respect: In the revised models, ratings were now significantly higher following cues with higher cue variance in pain trials (as in Yoshida et al., [2], although previously not replicated), but not in visual trials. The fact that controlling for block and trial effects altered the significance of these results suggests that the effects of cue variance are not very robust, and temporal effects were substantial in our data (indeed, there were significant block effects in both modalities, and significant trial effects in pain). We report the significant results but note that findings are not robust across different ways of handling covariates.

Results

"***Effects of cue variance on perception.*** *The cue variance affected perception of noxious stimuli, such that higher cue variance led to higher ratings (pain: $\beta = 0.046$, SE = 0.022 , $t_{(2845)} = 2.083$, p = .037), but there was no effect of the cue variance on ratings of visual stimuli ($\beta = 0.031$, SE = 0.021, $t_{(2875)} = 1.455$, p = .146). These results support the hypothesis that uncertainty is aversive particularly in the context of harmful stimuli [7], although this finding did not replicate in previous direct replication [37] and meta analysis [41]. These effects were, however, very small, and sensitive to analytical variability: without covariates for block and trial number (habituation/sensitization across time), the effect was significant in vision but not pain, consistent with marginal effects in both modalities.*" (page 13)

Finally, we now clarify the specific supplementary results on which we base conclusions described in the main text. For example:

Results

"Computational modeling strengthened the conclusion that most participants did not learn to ignore the cues during the task, since a comparison between five different models indicated a better fit of models without learning compared to models with learning (see Supplementary Results - Computational Modeling of Perception), in line with previous studies [9,64,66,73]." (page 15)

"In addition, computational modeling indicated that perception assimilates towards expected values more strongly in pain compared to visual perception, in line with the hypothesis that pain perception is more sensitive to contextual information (see Supplementary Results - Computational Modeling of Perception, comparison between w_p and w_v)." (page 15)

Comment 1.4

The authors presume that the only source of expectation is the fictitious ratings. While this is certainly an important source, as the experiment progresses, another significant source emerges—past experiences, which shape a different distribution of expected values. While the authors account somewhat for this by including a model where the weighting of the vicarious prior depends on prediction error (PE), they do not consider the possibility that expected pain could actually be a combination of recent pain experiences and social expectations. For instance, see (Jepma et al., 2018; Pavy et al., 2023). See also later points.

Response 1.4

Indeed, expectations depend on several factors, most notably the cues and the prior experience. While we do not assume that this is the only source of information influencing expectations, in the current manuscript we focused specifically on experimental effects of cue-based expectations. We believe this focus is appropriate because the exogenous randomization of the stimuli allowed us to estimate their causal effects, independent of other ongoing dynamic changes in experience. In addition, there are differences in the paradigm used here compared with other paradigms (e.g., Roy 2014, Jepma 2018, Pavy 2024 [3–5], others) that make dynamic expectancy effects driven by stimulus history less relevant. In many of these other studies, cues are stable indicators that acquire value over time as participants learn their meanings. Here, participants are instructed that each cue is unique and each trial is independent; there are no individual, stable cues that acquire value over time. While a complete model of endogenous, learning-driven fluctuations in expectations is beyond the scope of the paper, we do provide computational models that account for history (learning effects), which allows for endogenous variation in expectation updating based on past experiences, but find minimal evidence that history effects contribute significantly here. We now clarify this in the introduction:

Introduction
"Furthermore, although expectations are influenced by various factors, including cues, prior experience [40,66], and fluctuations in attention [67] among other factors, here we focus specifically on cue-based expectations." (page 6)

Comment 1.5

5. Variance, skewness, and cue mean were jointly manipulated, yet the authors tested them independently. The design does not ensure these variables are controlled for when testing one another, raising questions about the validity of the findings.

Response 1.5

The cue mean, variance, and skewness in our design are orthogonal, as we explicitly generated stimuli to vary them independently so we could estimate the effects of each in a fully crossed design, with equal numbers of trials in each combination of mean, standard deviation, and skewness. Moreover, the models

we have used tested them simultaneously (in the same model), and thus the effects of other distributional properties were controlled for when estimating the effects of each. Note that we also tested for and reported interactions, particularly between the cue mean and variance, as such an interaction is predicted by predictive coding theories.

We now describe this more clearly in the Results section, in addition to the previously included more detailed descriptions in the Methods section:

Results

"Expectation generation from distribution cues

In the expectation task, participants were presented with distribution cues with varying levels of mean (30, 40, 50, 60, or 70), standard deviation (5 or 12.5), and skewness (negative, positive, or symmetric). These three distributional properties were independently manipulated across cues in a fully crossed design. Following each cue, participants rated their expectations regarding noxious and visual stimuli (stimuli were not delivered during this task). The task included 360 trials overall (six repetitions of each combination of modality, cue mean, cue variance and cue skewness). The effects of cue mean, variance, and skewness on reported ratings were tested simultaneously with mixed effects models (one per modality) including predictors for each distributional property and their interactions, and controlling for block and trial number within each block." (page 8).

Minor Issues:

Comment 1.6

A) P6: Paragraph on effects of cue variance: The authors write "cue mean * modality interaction"—it is unclear whether the intention was to refer to cue variance instead.

Response 1.6

We thank the reviewer for pointing us to this mistake. It indeed should have stated "cue variance * modality interaction", not cue mean. Since we now test each modality separately (see Response 1.2), we do not test this interaction and thus this sentence was removed from the text.

Comment 1.7

P6: The sentence "Expectation ratings were significantly higher for lower cue variance in pain trials…but not in vision trials. In other words, participants expected more painful stimuli after more certain cues, but this was not true for noxious stimuli." It is unclear if "noxious" was meant to be "visual. Else, I do not understand what the last part of this sentence implies?

Response 1.7

We thank the reviewer for pointing us to this mistake; it should indeed have read "visual". We have now corrected this sentence:

Results

"*Expectation ratings were significantly higher for lower cue variance in pain trials (β = -0.045, SE = 0.014, t(58.1) = -3.16, p = .002), but not in visual trials (β = 0.003, SE = 0.013, t(66) = 0.25, p = .800). In other words, participants expected more painful stimuli after more certain cues only for pain stimuli.*" (page 8)

Comment 1.8

P6: By the time the effects of skewness are described, the operationalization of skewness and the predictors tested remain unclear.

Response 1.8

We previously described the operationalizations and models in the Methods section. We now added shorter descriptions in the results section, including description of the skewness operationalization and the predictors included in each model (see Response 1.1 and Response 1.5).

Comment 1.9

P7: The authors write: "In addition, expectation ratings were significantly higher in vision compared to pain trials. This result suggests that participants might have been biased by lower values particularly in the context of pain, or viewed themselves as less sensitive to pain compared to other participants." It is unclear how this conclusion was reached, as an alternative explanation seems equally plausible—that participants were more susceptible to higher ratings in the vision condition. The basis for determining the direction of the effect is not clearly explained.

Response 1.9

The reviewer is correct that both directions are equally plausible. We have now revised the manuscript to include separate models for pain and visual perception (see Response 1.2), and thus we no longer include interactions with modality, and the text the reviewer was referring to was removed from the revised manuscript.

Comment 1.10

P10: When the authors mention correlating parameters with personality traits, it would be helpful to mention the final sample size to assess the meaningfulness of these analyses.

Response 1.10

We have added the sample size to the results of these correlations (we also moved the detailed parameters to a Supplementary Table (see response 2.7).

Results

"*Finally, we tested whether the estimated k and b values correlated with state / trait scores based on questionnaires completed by the participants prior to the cued-perception task. For example, people with higher fear of pain, pain catastrophizing, or anxiety may focus more on cues predicting higher expected pain. However, no correlations between fear/anxiety-related measures (Fear of Pain score [69], Pain Catastrophizing score [70], and State-Trait Anxiety Inventory score [71]) and optimized k or b were significant for either modality (N = 45; all |r| <= 0.22 and all p >= .140; see Table S1).*" (page 10)

Comment 1.11

P10: The section on "The effects of cue-based expectation on perception" lacks sufficient information for the reader to fully understand this results section. Like how many intensity levels were presented and how the cue mean relates to participants' pain ratings.

Response 1.11

As noted in previous comments, we have now revised the results section and provide the main methodological details at the beginning of each subsection of the results. We hope this adds clarity.

Results

*"**The effects of cue-based expectations on perception***

In the cued-perception task (for a detailed description see Methods and Figure 1), participants viewed cues consisting of a distribution of 10 values (with a fixed mean value of 30 or 70, low or high variance, and negative, symmetric or positive skewness) followed by painful heat (47°C or 48°C) or flickering checkerboards (50% or 60% luminance contrast). This task included 144 trials overall (three repetitions of each combination of modality, stimulus intensity, cue mean, cue variance and cue skewness). The effects of stimulus intensity and cue mean, variance, and skewness on reported ratings were tested simultaneously with mixed effects models (one per modality) including these predictors and their interactions, as well as block number and trial number within block as additional numeric regressors (to control for temporal effects such as habituation and sensitization [72])." (page 12).

Comment 1.12

P11: The authors write:" Participants rated stimuli with higher cue variance as more intense (= 0.037, SE = 0.016 , t(5857) = 2.320, p = .020). This finding is consistent with Yoshida et al., (7), who suggested that uncertainty is aversive in the context of pain and thus lead to higher pain ratings, although a direct replication did not find this effect (30)." In the same paragraph is later stated that these effects only held for visual stimuli, undermining this initial conclusion.

Response 1.12

Following comments 1.3 and 1.13, we added regressors to the models to control for trial and block effects. As a result, higher cue variance is now significantly leading to higher ratings of pain, but not visual contrast. As discussed in Response 1.3, above, we report the findings but also note that the effects are small and they are not robust across different modeling choices. In addition, we have included extended caveats in the Discussion: Discussion:

"Bayesian accounts of brain function also make specific predictions about the precision of expectations. More certain (precise) cues should have stronger effects on perception [5,6,30]. Some previous studies have provided support for this hypothesis by using the precision of stimulus history as a proxy for predictive precision [25,34]. The kinds of cues with distributions we used here offer particular advantages in studying cue precision, as the cue mean, variance (i.e., inverse precision) and other properties (e.g., extreme values) can be independently experimentally manipulated across cues. Studies using this type of cues have found results that contradict predictive processing accounts by finding null effects of cue precision [37] or even direct effects of predictive uncertainty on increased pain perception [7]. In the current study, cue precision effects supported Bayesian predictive processing accounts in the visual modality, by showing a cue mean x variance interaction. In contrast, more uncertain cues led to higher ratings of painfulness of noxious, but not visual, stimuli, suggesting that uncertainty may be aversive in the context of pain, as previously suggested by Yoshida et al. [7]. However, these findings were previously not replicated [37] and not supported by a recent meta analysis [41]. Here, the effect sizes were small and of marginal significance in pain, and not robust to methodological variations in the covariates included." (page 25)

Comment 1.13

P12: There are strong block effects (likely due to thermode repositioning) and within-block habituation effects in the pain data. It is unclear whether the authors controlled for these effects in their models, and if not, how these factors might have influenced the other findings. Additionally, in other parts of the analysis, the authors mention "trial effects," but it is not clear what exactly this refers to, requiring further clarification and why it was included in these analysis and not other analysis.

Response 1.13

Trial effects model variation in outcomes (pain or visual intensity) over time within block, thus estimating and controlling for sensitization/habituation. Within-block habituation is a common finding across studies of pain, as within-block effects involve repeated stimulation on the same skin site (e.g., Jepma et al. 2014), though our experimental design ensured that such effects are orthogonal to main effects of cues (cue mean, variance, and skewness). Regardless, we control for trial and block effects in all analyses, and have revised our results and conclusions accordingly (see Response 1.3 and Response 1.12).

Comment 1.14

P13: The authors write: "Computational modeling strengthened the conclusion that most participants did not learn to ignore the cues during the task (see Supplementary Results - Computational Modeling of Perception), in line with previous studies (9,60–62)." It is unclear what exactly this conclusion is based on. Was this conclusion drawn from the model that incorporated the influence of prediction error (PE) on the weighting of cue expectations? As I understand it, this model was not preferred for the majority of individuals nor based group level model comparison, so it remains unclear how the authors arrived at this conclusion. Further explanation is needed.

Response 1.14

This conclusion was based on the finding (presented in the Supplementary Information) that for most participants, the best fitting model was a model that did not include a learning component. This indicates that cue effects persist across time and participants do not learn that they are irrelevant, as shown in other analyses as well (they are significant even at the end of the experiment). We now clarify this in the revised text:

Results:
"*Computational modeling strengthened the conclusion that most participants did not learn to ignore the cues during the task, since a comparison between five different models indicated a better fit of models without learning compared to models with learning (see Supplementary Results - Computational Modeling of Perception), in line with previous studies [9,64,66,73].*" (page 15)

Comment 1.15

P.21: The authors write: "Together with the assimilation towards predicted values in higher-level systems, our results largely support this working hypothesis, although we do not directly test encoding of different types of PEs (80)." It is unclear why the authors did not test this, as it could have been easily done. An expectation distribution combining both the vicarious information and participants' past pain ratings could have provided much more meaningful insights into how different types of expectations and information jointly shape pain experiences (see, for example, Jepma et al., 2018; Pavy et al., 2023), where a similar dynamic approach was taken. This would enhance the understanding of the interplay between social and personal expectations in pain perception.

Response 1.15

Since we have used fixed cue mean value and stimulus intensities, the estimation of the subjective value of each stimulus for the specific participant in our data is not ideal. Furthermore, we did not collect expectations ratings during the cued-perception task. Thus, while providing some relevant evidence, a more direct and in depth test of the effect of different types of PEs (negative, positive, absolute, etc.) is beyond the scope of the current study. As noted above, though we have focused on history-based learning models

in other work, our main focus here was to assess the effects of experimentally randomized and orthogonalized independent variables (cue mean, variance, and skewness).

Comment 1.16

The authors strongly emphasize the Yoshida findings throughout the paper, but a recent meta-analysis (Pavy et al., 2024) has clearly demonstrated that uncertainty does not consistently lead to more pain. It would be advisable to portray this relationship in a more nuanced way, reflecting the broader evidence and avoiding overemphasis on a single set of findings.

Response 1.16

We agree with the reviewer and now put this finding in the context of the broader evidence (but also our revised results, which do find increased pain ratings following more uncertain cues in pain). In addition to the nuance when presenting results (Response 1.3) and discussion (Response 1.12), we have taken care to address the previous lack of replication in the introduction as well:

Introduction
"*Thus, Bayesian models predict an interaction between the expected value and its precision, such that more certain expectations (prior beliefs) should affect perception (posterior beliefs) more strongly. This hypothesis has been supported by studies on pain perception showing stronger placebo analgesia in individuals with more precise treatment expectations [25,33], and greater assimilation of perceived pain towards expectations with higher cue precision [34,35]. Conversely, other studies have found that lower precision or predictability (higher uncertainty) leads to more pain, putatively because uncertainty is aversive in the context of potential harmful stimuli [7,36]. Some other studies did not replicate these effects and even found instead that more certain cues led to more pain [37–41]. Finally, a recent meta analysis of 70 studies did not find an effect of predictability (including uncertainty about timing, duration, or location) on pain perception [41]. Instead, meta-regression analyses showed that pain was higher for whichever condition (unpredictable vs. predictable) elicited higher pain expectancy and state anxiety, implying that expectations may confound predictability manipulations in some studies.*" (page 3)

Comment 1.17

P22-23: The authors write: "If participants over-weight extreme high-pain values—e.g., attend most to the most threatening potential values—that might explain why Yoshida et al. found increased pain with high-variance cues (7) while other studies have not found a main effect of uncertainty on pain (30,35). Here, we found that perceptual ratings were drawn towards larger extreme values, but this effect, like the effects of the cue variance, was significant mostly in visual perception and not in pain perception." There are two main issues with this:
i. It seems inconsistent to use this effect as an explanation for pain findings when it only held for visual stimuli in your own research. If the effect was not observed in the pain domain, it weakens the relevance of this explanation for the pain findings.
ii. The proposed explanation for Yoshida's findings does not account for the broader literature. A well-powered replication study (Zaman et al., 2017) found no effect of uncertainty on pain perception despite using the identical paradigm, making the assumption that skewness drove

their results unlikely. This needs to be addressed to provide a more balanced interpretation of the findings.

Response 1.17

We have now revised this text as well as other parts of the manuscript. First, as described in response 1.3, our revised results do replicate Yoshida's et al. findings in that participants report slightly higher pain with higher cue variance, though the effect is small (as we note in the revised manuscript). Second, we now clarify throughout the manuscript that their findings were not replicated in a previous direct replication study and not supported by a recent meta analysis (see also response 1.19). Finally, the computational models of how cues are weighted and integrated do not suggest a mechanism that supports Yoshida's findings, as we now clarify (see Response 1.19 below).

Comment 1.18

P23: The authors write: "Finally, the persistent effects of the nonreinforced cues in both pain and visual perception might represent modulation of early perceptual processes, modulation of perceptual decision-making." The repeated mention of "nonreinforced cues" is confusing (how I understood it they always received a stimulus during the cued-perception tasks) and suggests that I did not fully understood the task, even after reviewing the methods section multiple times. Further clarification is needed regarding the use and definition of these nonreinforced cues to ensure the task design is fully comprehensible.

Response 1.18

We appreciate the need to clarify the task, and have revised the manuscript to better define the cues and motivate the choice of such cues. We have conducted studies with individually calibrated stimulus intensities and informative cues that signaled high or low future stimulus intensity (e.g., Atlas et al. 2010). Our view is that such cues can work partly through cognitive expectancies, and partly because of classical conditioning in lower-level nociceptive brain circuits. E.g., with enough training, the spinal cord alone can learn to respond to pain-predictive cues [6]. This is why we chose here to make the actual stimulus intensity independent of the cues. In past work cited in the response below, we have found that surprisingly, even cues that are not predictive of actual stimulus intensity can exert strong and persistent effects on pain reports, and on pain-related autonomic responses as well (e.g., [7]). We interpret these effects as pure effects of cognitive expectancies without the possibility of classical conditioning in nociceptive systems.
We have clarified this in the introduction:

"*Here, we test how expectations are generated from distribution cues with varying levels of mean, variance, and skewness (and thus the presence and direction of outliers), and how they affect subsequent perception of visual and painful stimuli (for the study design and illustration of the cues see Figure 1 and Methods). The cues were not externally reinforced (i.e., not predictive of subsequent stimulus intensity), and thus were informational but not subject to associative learning (i.e., they were not classically conditioned). They also did not depend on the participant's individually calibrated intensities, similar to several previous studies [8,10,64,65] (we note that in some other studies, cues were based on the individual's psychometric curve*

[7,9,37]). Such cues allow us to focus on effects of cognitive predictive information, rather than classical conditioning, which can occur when cues are associated with different levels of subsequent stimulus intensity and are thus truly informative about future intensity.“ (page 6)

Comment 1.19

P23: The authors write: "Beyond the assimilation to the mean value, reported expectations were also biased towards extreme values in both modalities, and specifically smaller values in pain." This effect seems to directly contradict the authors' presumed explanation of Yoshida’s findings. If skewness is driving the results, pain ratings should be lower in high variability conditions, which was not observed. This inconsistency needs to be addressed, as it undermines the author’s explanation linking extreme value biases with Yoshida’s findings.

Response 1.19

The reviewer is correct, and we have now revised the manuscript to clarify that our findings do not support the original hypothesis that extreme values may explain the inconsistency of previous studies regarding the aversiveness of uncertainty in the context of pain. For example:

Results:
“*Taken together, the behavioral and computational results of the expectation task suggest that while expectations strongly assimilate towards the mean cue value (Figure 2A), participants overweight extreme cue values in both modalities (Figure 2B and Figure 3), and demonstrate an optimism bias specifically in pain, relying more on low than high extreme values (Figure 3). Such effects were not considered in most previous studies, but contrary to our hypothesis, they do not explain previous findings of uncertainty aversiveness in pain based on unintentional manipulation of extreme values when manipulating variance, since participants (at least on our sample) were more attentive to lower rather than higher extreme pain values.*” (page 12).

Discussion:
“*We also tested the effects of extreme cue values, and how participants weight predictions of upcoming pain and visual contrast across the distribution from low to high values, which has been studied in the context of magnitude judgments [50,51] but not, to our knowledge, in pain. We hypothesized that over-weighting of extreme values might account for previous inconsistent findings in the context of the effect of cue variance on pain, if participants over-weight extreme high-pain values (e.g., attend most to the most threatening potential values) and extreme values were unintentionally manipulated with the variance. However, this hypothesis was not supported by our data, since (1) participants in our sample were more attentive to low, rather than high, pain value when generating expectations; (2) higher cue variance led to slightly higher pain ratings even when outliers were orthogonal and controlled for; and (3) perceptual ratings were drawn towards larger extreme values only in visual (and not pain) perception.*” (page 27)

Comment 1.20

P26: The authors write that 25 stimuli were presented in a randomized order, for a total of 50 stimuli. It is unclear from this sentence how many pain stimuli were actually presented—25 or 50? Later, it appears that 25 stimuli were used, based on 5 different intensities and 5 repetitions. This should be clarified

Response 1.20

There were two blocks, one for pain and one for vision, with 25 stimuli per block. We have now revised the text to clarify:

Methods
"*The task was split into two blocks, one pain block and one vision block. In each block, 25 stimuli were presented in randomized order.*" (page 31)

Comment 1.21

P27: Two levels of stimulation intensity are mentioned. Were these levels fixed or calibrated to the individual's pain threshold, corresponding to specific pain ratings? This information is essential for understanding the methodology.

Response 1.21

The stimulus intensity levels were fixed, and not calibrated to the individual's pain threshold (see also response 1.1). We now clarify this in the revised manuscript:

Methods:
"*In this task, stimulation modality (heat vs. visual) and two levels of stimulation intensity (47°C or 48°C, or 50% or 60% luminance contrast) were combined with different rating cue distributions similar to those used in the expectation task.*" (page 33)

Results:
"*In the current study, we have used fixed stimulus intensities and cue mean values (i.e., these values were not calibrated to each participant), which could potentially result in large discrepancies for some participants, inducing such boundary effects. To test for such effects, we performed additional analyses similar to those of Hird et al. [43]...*" (page 16)

Discussion:
"*Several limitations should be considered in the context of the current findings. First, we have used cues that were not predictive of actual stimulus intensity (i.e., non-reinforced), with fixed mean levels that did not depend on participants' individual psychometric curves (i.e., how intense the stimulus is expected to be perceived by the specific individual), and fixed stimulus intensity levels (that were not calibrated to each participant).*" (page 28)

Comment 1.22

P28: The authors write: “The rating cues were drawn from distributions with two means ([30, 70]), two standard deviations ([5, 12.5]), and three levels of skewness (negative skew, symmetric, positive skew).” Unlike Yoshida et al., where the vicarious ratings were directly related to the participants' expected pain ratings based on their psychometric function, it seems that here the ratings were unrelated. If this is correct, the authors should document how much the cued expectations differed between subsequent ratings, and whether large differences might have led to reduced credibility of the cued information.

Response 1.22

As explained in response 1.1, we indeed used fixed cue means, and we found that these cues produced consistent cue effects (here and in previous studies). Additionally, we did not find evidence for boundary effects in the current study, and thus there does not seem to be an issue of the cues’ believability.

Comment 1.23

P29: Please specify, for each predictor, whether it was categorical or continuous, and whether trial or block effects were included in the analysis.

Response 1.23

We now fully describe all regressors (in the Results, and not only Methods section), and their class. We also added trial and block effects and now describe when they are included in the models. See response 1.3.

Comment 1.24

P30: The description of the 5 different computational models is surprising, given that all the relevant analyses are presented in the supplementary information. Consider moving the model descriptions to the supplementary materials as well for consistency.

Response 1.24

We moved the model descriptions to the Supplementary Materials.

In conclusion, while the manuscript addresses an interesting topic, several aspects of the design, analysis, and interpretation need clarification to improve the clarity and robustness of the study. I enjoyed reading the manuscript and thinking about it. I hope the authors find my suggestions helpful.

Sincerely,
Jonas Zaman

References

Jepma, M., Koban, L., van Doorn, J., Jones, M., & Wager, T. D. (2018). Behavioural and neural evidence for self-reinforcing expectancy effects on pain. Nature Human Behaviour, 2(11), 838–855. https://doi.org/10.1038/s41562-018-0455-8

Pavy, F., Zaman, J., Van den Noortgate, W., Scarpa, A., von Leupoldt, A., & Torta, D. M. (2024). The effect of unpredictability on the perception of pain: a systematic review and meta-analysis. Pain, 165(8), 1702–1718. https://doi.org/10.1097/j.pain.0000000000003199

Pavy, F., Zaman, J., Von Leupoldt, A., & Torta, D. M. (2023). Expectations underlie the effects of unpredictable pain: a behavioral and electroencephalogram study. Pain. https://doi.org/10.1097/j.pain.0000000000003046

Zaman, J., Vanpaemel, W., Aelbrecht, C., Tuerlinckx, F., & Vlaeyen, J. W. S. (2017). Biased pain reports through vicarious information: A computational approach to investigate the role of uncertainty. Cognition, 169, 54–60. https://doi.org/10.1016/j.cognition.2017.07.009

Reviewer 2

This paper addresses multiple different conflicts in literature and thoroughly discusses them through experiments, models and neural analysis:
- They define expectation experiment to understand how people's expectations are shaped by varying levels of mean, variance, and skewness
- This experiment, specially the skewness experiment allowed them to investigate the influence of presence and direction of outliers
- They further investigated the influence of the above on pain and visual perception
- Additionally exploring the pain and visual domain allowed them to identify similarities and differences of influence of expectation on perception in different modalities
- Finally they investigated how neural responses during pain and visual perception are affected by the different properties of the cue and by the cue-based expectations
The paper does a great job of detailing the models and the experiment and elaborately connect results to the literature in the discussion section

There are some questions that I need some clarification on and have some small comments for improvements:

Comment 2.1

- Introduction first paragraph says: "However, which brain signals assimilate to expectations, perhaps forming a neural substrate for perception, and which encode PEs and thus drive learning, is a topic of active investigation." I would like authors to add some appropriate citations

Response 2.1

Thank you. We have now added several citations supporting this statement. There are a large number to choose from, and we hope that our selection has covered some of the important ones the reviewer had in mind. The revised text is below:

Introduction:
"*However, which brain signals assimilate to expectations, perhaps forming a neural substrate for perception, and which encode PEs and thus drive learning, is a topic of active investigation [5,22–30].* "
(page 3)

Comment 2.2

- Introduction says "may stem from differences across experimental designs …." It would be good to summarize the follow-up statements into a table and also additionally mentioning how their work resolves/addresses some of them in a new column. Since this paper has multiple points to make, a summary discussion is easy to get the take away message.

Response 2.2

We thank the reviewer for this suggestion, and have now added such a table to the main text:

Introduction:
"*The contradicting findings regarding the effect of the cue certainty on pain perception [42] may stem from several factors, such as experimental design and sensory modality. For examples and descriptions of how we address them in the current study, see Table 1.*" (page 3)

Table 1. Factors that could explain previous contradicting results.

Factor	Examples	How previous studies differ	How the current study addresses it
Experimental design	Precision manipulation	Previous studies may have unintentionally manipulated additional statistical properties of cues (e.g., outliers, skewness) when altering precision.	This study systematically tests the independent effects of cue mean, variance, and skewness to isolate their contributions.
Sensory modality	Pain vs. other stimuli	Uncertainty may be more aversive in pain studies due to its threat value, leading to different effects across sensory modalities. Most studies focus only on one sensory domain.	This study employs a within-subject design to compare uncertainty effects in both pain and visual perception.
Participants	Individual differences, trust in cues	Variability in how participants perceive uncertainty (e.g., aversiveness), their self-perception (e.g., pain sensitivity), and trust in provided information can lead to mixed results.	Our sample is based on a specific population like previous studies, and we do not directly manipulate individual differences, but some are measured (e.g., state anxiety, fear of pain, pain catastrophizing). Moreover, the study accounts for trust in the cues by tracking cue effects over time to ensure their believability.

Comment 2.3

- Introduction says "Furthermore, this kind of nonlinear weighting might depend on the sensory modality …" : It would be good to provide some reference and better motivate the study of pain modality. Additionally I would like to better understand a summary of what the literature says for other modalities and how pain and vision compare to that. I also did not find a clear motivation to using these two modalities

Response 2.3

We thank the reviewer for this comment, and recognize the need to better motivate the choice of pain and vision as sensory modalities in the current study. The motivation for this study originated from recent predictive coding theories and findings, and inconsistent findings regarding the role of uncertainty in perception, and particularly pain perception. Pain is a unique modality in that it is tied to threat, which may induce pain-specific properties such as aversiveness of uncertainty (as suggested before), or amplified influence of extreme values (threat or safety signals). We chose the visual modality as the comparison modality for three main reasons: First, predictive coding theories originated from studies on visual perception [8], and visual perception is extensively studied in the context of predictive coding and expectations [9]. Second, the visual modality is affectively neutral and thus allows us to test for unique characteristics of negatively compared to neutrally valenced modalities. Third, nonlinear weighting has been previously demonstrated in vision, but has not systematically studied in pain. In addition, we thought that vision might be less subject to large influences of cues on perception than pain, though our findings here suggest there are robust cue effects in both.

As for other modalities, predictive coding has been studied in audition, and like vision and pain, there are findings supporting it but also many open questions regarding the role of uncertainty, where in the brain different properties are coded, and more [10,11]. Nonlinear weighting has also been shown in audition, for example in the study demonstrating nonlinear weighting when determining which series of numbers was on average larger, this effect was demonstrated in both vision and audition [12]. All in all, the literature on nonlinear weighting of cues or stimuli is limited, and by investigating both pain and visual perception we were able to test modality-general and modality-specific properties of cue-based expectation generation and their effect on perception.

We have now revised the introduction to better motivate the choice of the pain and visual modalities

Introduction:
"*... participants may instead attend to the highest or lowest values, depending on the task and their predispositions (e.g., if some participants find pain to be threatening and are vigilant for high-pain cues). Such effects are not taken into consideration in most studies testing Bayesian accounts of perception, but there is evidence that they can be important. For instance, a previous study in visual perception has demonstrated overweighting of inliers (values closer to the mean) [50]. Such effects may also be sensitive to the precise demands of the task. For example, when asked to determine which series of numbers was on average larger (via visual or auditory perception), people have been found to overweight outliers (extreme values), specifically larger values [51]. Furthermore, this kind of nonlinear weighting might depend on the sensory modality, and has not been studied in the context of pain perception. If attention to extreme values is related to vigilance for potential threat, people might weight them more strongly for pain than for other, safer modalities. This could, in principle, explain previous findings of higher pain ratings following less precise cues [7]: If people are biased towards maximal pain values ("danger signals"), and higher variance entails larger maximal values, then this effect is expected.*" (page 4)

"*By comparing pain and visual perception, we identify common and distinct mechanisms for expectation formation and modulation of perception for stimuli that are potentially harmful (painful) and those that are non-harmful and affectively neutral (visual contrast). The visual modality was chosen because predictive*

coding theories originated in this domain [68] and have been extensively studied within it [5]. Additionally, as noted earlier, nonlinear weighting has been demonstrated in visual perception." (page 6)

Comment 2.4

- I am curious to understand I am trying to understand how the expectation generation is different from updating the parameters of prior distribution in Bayesian inference, particularly is this any different from tuning/learning hyperparameters in Bayesian statistics?

Response 2.4

These two processes indeed share conceptual similarities, as both involve adjusting beliefs in response to incoming information. If perception strictly follows Bayesian principles, then participants' expectations are indeed reflected in the prior distribution, and expectancy updates would reflect a shift in priors. Hyperparameters can reflect second-order beliefs, e.g., how quickly priors should change to track volatility in the characteristics of the environment, but are not part of our modeling approach here. One of the main goals of our study was to test whether perception follows Bayesian principles, across modalities. For example, we test the role of uncertainty of information (here, the variance of the cue values), which according to Bayesian inference should modulate the effect of expectations on perceptual judgments (a prediction that is only partly supported by our findings, particularly in subjective reports of visual perception, but not in pain perception or brain responses in both modalities). Moreover, predictive coding and other Bayesian models, to the best of our knowledge, do not directly address how expectations (prior beliefs) are generated from multiple cue values based on the distribution of the cue (e.g., outliers), and thus we focus on this process, in addition to the influence of cue-based expectations on perceptual judgements. Finally, as noted in response 1.4, we focus here on cue-based expectations (and not other sources of information used to shape expectations). We now clarify this in the manuscript by revising the title and other mentions of "expectation generation" to "cue-based expectation generation".

Comment 2.5

- The authors have a computational model for weighting of cue values, but from the methods I am sure I understand how they made sure the weights were not biased by the outliers? logistic regression is supposed to be influenced by outliers, so how did the authors ensure the overweighting of outliers was not an artifact? ref: results section "We found that participants significantly over-weighted outliers in both modalities.."

Response 2.5

Thank you for the opportunity to clarify. There are two kinds of outliers here: Outliers in the cues, i.e., when one of the 10 cue lines was very different from others, and outliers in the traditional statistical sense, i.e., large residuals presumed to arise from a different error distribution than other observations. Our outliers are the former type here, but these "outlier" cues were frequent in our design, and so are not statistical anomalies in the traditional sense that would pose a problem for model fitting. The computational model of the cue-based expectation generation was developed to test whether people differently weight cue outliers (lines far from the others in a cue) vs. inliers (lines similar to others in a cue), and was inspired by the model of Spitzer et al., 2017 [12]. We quantified the over-weighting of outliers based on the power term included in the model, with the free parameter k (see Figure 3A). This was not tested with logistic regression, but rather an additional logistic term was used to model overweighting of smaller vs. larger values. Thus, the test of weighting outliers was not dependent on a logistic regression. The finding that participants overweighted extreme values was based on Wilcoxon signed rank test revealing that k was larger than 1 (i.e., outliers were overweighted) in both modalities. We visualized data distributions and used nonparametric tests (i.e., Wilcoxon) to assess and minimize the influence of statistical outliers, and we are confident that our results are not driven by those.

Comment 2.6

- Figure 3A, I am confused by the end points of the plot for k=100. In Figure 3B, I am worried about Simpson's paradox, can the authors comment on that a little. Figure 3C, this is across all subjects, correct? Are there any errors bars? Is the result significant? Also in general there is a lack of figure reference in text in conclusive paragraphs in results section. The statement "participants overweight extreme cue values in both modalities, and demonstrate an optimism bias specifically in pain, relying more on low than high extreme values." is that based on Figure 3 c alone? Like mentioned before, additional figure references in such conclusions will help readers.

Response 2.6

We appreciate the need to clarify the plots in Figure 3. When k = 100, outliers in both ends (low or high) are extremely overweighted, and thus their weights go up substantially in Figure 3A.
In Figure 3B, illustrating the correlation between expected and predicted expectations, the correlation is indeed enhanced by the five levels of cue mean, however, when computed within each level of cue mean level, all correlations are still very significant. We now clarify this in the main text:

Methods
"*Thus, the model computes an expectation value for each cue. The model was fitted to all trials (cues) of the expectation task of each participant... We computed Pearson's correlation between observed (reported) expectations and model-based predicted expectations. Since the five different levels of cue mean contributed to the correlation and could artificially enhance the correlation between observed and predicted value, we also computed the correlation within each cue mean level.*" (page 37)

Results

"The expectation model fit the data well overall, with an average correlation between predicted and empirical expectation ratings across participants of Pearson's r = 0.945 (SD = 0.032) for pain and r = 0.928 (SD = 0.074) for vision, and an average root mean squared error (RMSE) of 5.904 (SD = 1.668) for pain and 6.415 (SD = 2.545) for vision (on a rating scale of 0-100). The Pearson's correlation was lower, but still high and very significant, when tested separately within each level of cue mean (range of mean correlation across participants, across all cue mean levels: pain 0.344-0.424; visual perception 0.188-0.375, all ps < .001). This indicates that participants tracked variation in cues accurately, and that the model predicted their responses well and performed generally better in pain than in vision (Figure 3B)." (page 10)

As for Figure 3C, it illustrates the weight function of the median optimized *k* and *b* values across participants for each modality, with an arbitrary choice of equally distributed values between 20 and 60. We have now added the weight functions of individual participants to the plot:

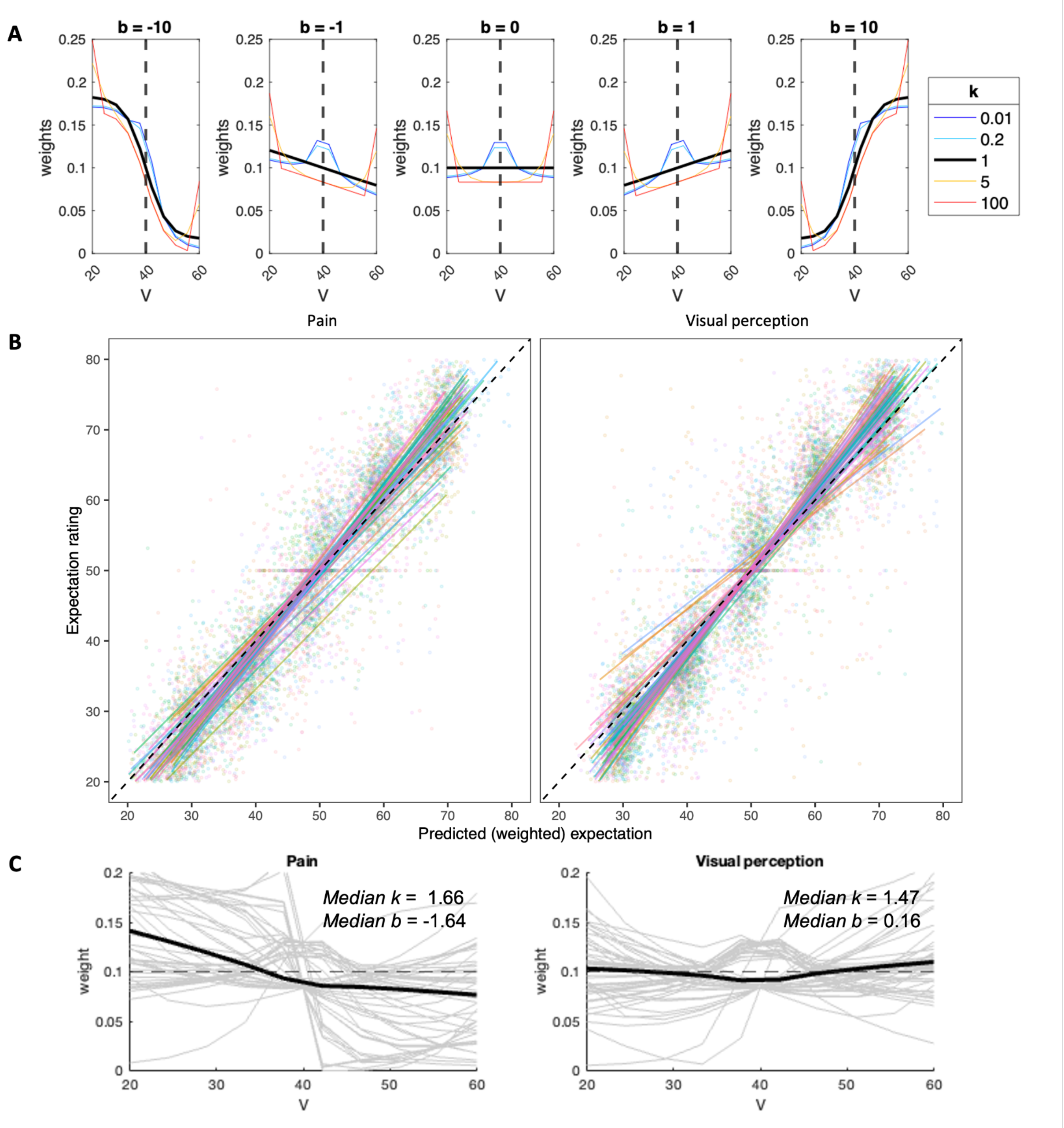

We have now also added more references to the relevant figures where conclusions are stated. As for the conclusion that "participants overweight extreme cue values in both modalities, and demonstrate an optimism bias specifically in pain, relying more on low than high extreme values", this conclusion is based on the results of the computational models of expectation generation, indicating that the median k value across participants is significantly larger than 1 in both modalities, and that the median b values across participants is significantly smaller than 0 in pain (and not different than 0 in visual perception). As indicated above in the current response, Figure 3C relates to these results as it illustrates the weight function across participants, but the conclusions are based on the statistical tests that are now presented in a figure.

Comment 2.7

- The authors put too much parameter details in the text which is hard to follow when reading, it would be great to have references in form of tables. The authors already have multiple tables in the supplementary and they is easy to read and follow.. Example in Results section: "However, all correlations between the available measures in our sample (Fear of Pain score (56), Pain Catastrophizing score (57), and State-Trait Anxiety Inventory score (58)) and optimized k or b for each modality across participants, were not significant …." has too much parameters added which is hard to follow and conclude the findings.

Response 2.7

Following comments by other reviewers, we have now reduced the number of analyses by only focusing on separate models for pain and vision (see response 1.2). In addition, we now move more parameters to Supplementary Tables, including the results of the questionnaires that the reviewer mentioned were difficult to follow when in-text:

Results:
"*Finally, we tested whether the estimated k and b values correlated with state / trait scores based on questionnaires completed by the participants prior to the cued-perception task. For example, people with higher fear of pain, pain catastrophizing, or anxiety may focus more on cues predicting higher expected pain. However, no correlations between fear/anxiety-related measures (Fear of Pain score [69], Pain Catastrophizing score [70], and State-Trait Anxiety Inventory score [71]) and optimized k or b were significant for either modality ($N = 45$; all $|r| <= 0.22$ and all $p >= .140$; see Table S1).*" (page 10)

Table S1. Results of correlation tests between estimated *k* and *b* values and state / trait scores.

Questionnaire	Parameter	Modality	Pearson's r	p values
Fear of Pain	k	Pain	0.16	.284
		vision	0.04	.800
	b	Pain	0.22	.140
		vision	0.03	.825
Pain Catastrophizing	k	Pain	-0.17	.267
		vision	0.09	.553
	b	Pain	0.08	.582
		vision	0.06	.705
State Anxiety	k	Pain	0.15	.320
		vision	0.13	.402
	b	Pain	0.05	.734
		vision	-0.02	.905

Comment 2.8

- In Figure 4C I am not sure I understand why the plot looks zagged. Does the error bar represent across subject variability?

Response 2.8

Error bars in Figure 4C indeed represent variability (standard errors) across participants. They look zagged because there is variability across trials, particularly in pain where there is habituation across trials within the same block (stimuli delivered to the same skin site) and sensitization between blocks (when moving the thermode to a new skin site; dashed lines separate blocks), as has been shown in previous research [13]. See also response 1.3.

We explain this in the figure legend:

"***Figure 4. Behavioral results, cued-perception task.*** *... **(C)** The effect of cue mean (color) on pain and visual contrast ratings over time, for low vs. high stimulus intensity (columns), in pain and visual perception (rows). Error bars represent standard error of the mean across participants. The lines represent the linear fit, and the shading represents 95% CIs of the linear fit. Vertical dashed lines separate between different blocks (note that a new skin site was used for each block, and thus the increase in the averaged pain rating for the first trial of each block stems from site-nonspecific sensitization and site-specific habituation [72]).*"

Comment 2.9

- I would also like to understand within subject variability of the expectations and perceptions, specifically if it is more for vision versus pain?

Response 2.9

In the expectation test, the within subject variability is indeed numerically larger for vision compared to pain, but this difference is not significant (pain: M = 16.8, SD = 1.56; visual perception: M = 17.2, SD = 2.09; t_{44} = -1.846, p = .072). In the perception task, the within subject variability is significantly larger for pain compared to visual perception (pain: M = 19.5, SD = 4.18; visual perception: M = 14.5, SD = 3.57; t_{44} = 7.259, p < .001). Note that these results are robust to performing a Wilcoxon test rather than a paired t test, and also to computing within subject variability as the average SD within each cue mean level (for the expectation data) or stimulus intensity (for the perception data).

Comment 2.10

- Minor: Is there a typo in this sentence? "This result suggests that participants might have been biased by lower values particularly in the context of pain, or viewed themselves as less sensitive to pain compared to other participants "

Response 2.10

Following comment 1.2, we have now removed this result (and its interpretation) from the revised text.

Reviewer 3

Summary and general evaluation:
The manuscript at hand explores the significant topic of how expectations influence perception in pain research through a computational approach. While it is clear that expectations have an influence on the perception of pain, the effect of its associated uncertainty is still not investigated in detail and led to conflicting evidence in the research literature. One issue that might be at the heart of it is the influence of outliers and skewness of cues that could lead to a biased way of building up expectations. The used paradigm presents distributions of cues as pain intensity and visual contrast ratings by previous participants and proves to be extremely useful to investigate such effects. The authors use both conventional linear mixed models and an innovative computational model to incorporate these influences on expectations to explain their expectation rating data. They reveal some interesting aspects that have not been explored previously, for example that participants over-weight extreme values in the cue distribution when they construct their expectations. In detail, they found a domain-independent over-weighting of outliers and a pain-specific over-weighting of values smaller than the cue mean value like an optimism-bias. This is a real strength of the manuscript, as it provides valuable starting points for further investigations into context, paradigms and populations in which such results would persist or differ, e.g., clinical populations with chronic pain.

In addition, the authors explore the subsequent effect on the perception process. In a separate experiment session, the participants received similar visual cues followed by real stimuli and had to rate the intensity of their percept. They also use a linear mixed model to fit the responses and find that it is mainly the used intensity and the cue mean that influence these subsequent responses. Only for visual stimuli they find some additional effects of cue variance and skewness on perception. The authors then check for stimulus intensity and cue effects in the brain by testing the concurrently recorded fMRI data for such trial-wise patterns. Whereas their previously developed neuromarker for pain (NPS) mainly activated in direction of stimulus intensity, their neuromarker for intensity independent processed captured the influence of the cue mean, which nicely fits expectations about the nature of these biomarkers. They add analyses for different ROIs to describe the effect of the cues on different perceptual processing stages. Additionally, they present a computational perceptual model that builds upon the expectation model but only refer to its results in the supplement. Overall, the figures clearly convey their messages and the manuscript is overall well written. The computational models are explained in detail and simulation figures enhance the understanding of their functionality. The authors provide additional data and code in their github repository that seems well maintained and accessible. The manuscript is generally properly placed in the context of the literature. The manuscript is of high value and clear importance especially to the pain community as predictive processing theories need to be formally tested using appropriate models before they find their way into clinical applications. I have a few issues I would like to see addressed before I could recommend the manuscript for publication (sorted from more major to more minor points).

Comment 3.1

1. The most severe point that hindered my understanding and evaluation of some of the results was the lack of description of the used intensities in the perceptual task. Whereas in the stimulus intensity task five different temperatures/contrasts are used, the authors describe the use of two intensities in the perception task but as far as I could tell, they do not describe how these were chosen. Depending on this, the evaluation of results in this task could drastically change. Predictive processing theories that predict an integration of cue information into the perception only hold true if the discrepancy between cue and stimulus information is not too strong. A highly discrepant stimuli will have a weaker influence on the perception as previously demonstrated by Hird et al., 2019, Nature Scientific Reports.
It would also help in evaluating how "threatening" the stimuli really were, as this could potentially influence how large cue values are interpreted. Knowing how far apart the two different intensities are would also help to evaluate how "ambiguous" the pain stimuli really were, as the authors mention several times that they are more ambiguous than the visual stimuli, yet do not provide information on that. This would also change how informative the cues then were to the participants. If the used intensities are that far apart that it becomes clear to many participants that they are actually just deciding between two competing hypotheses they might abandon the cues altogether, especially as they are not informative about the upcoming stimulus as the authors describe. It would be nice to not only see these points addressed but also to maybe see results about that in the figure 4 about the perception task. It would be great to see for example separate lines for the different intensity levels in 4C to see both the effects of stimulus intensity and cue mean as these effects are the strongest in these analyses.

Response 3.1

We thank the reviewer for these important comments. We indeed mistakenly omitted the intensity levels in our original manuscript, and now properly describe them (see also Response 1.11):

"*In the cued-perception task (for a detailed description see Methods and Figure 1), participants viewed cues consisting of a distribution of 10 values (with a fixed mean value of 30 or 70, low or high variance, and negative, symmetric or positive skewness) followed by painful heat (47°C or 48°C) or flickering checkerboards (50% or 60% luminance contrast).*" (page 12)

As explained in Response 1.1, the cue mean values and stimulus intensities were not calibrated for each individual participant. Nonetheless, we do not find any boundary effects in our data (see Response 1.1). Rather, our results indicate that the effect of the cue mean decreased but yet persisted throughout the task, and was still significant in the last block, for both modalities.
In our experience here and in other studies, the intensities we chose (*47°C* and *48°C)* are clearly painful but are relatively close and thus ambiguous. They are moderately discriminable across trials, and subjectively participants do not report knowledge that there are discrete intensity categories. We have now revised Figure 4C to separately illustrate the effect of the cue within each stimulus intensity and modality. As can be seen in the revised figure, the ratings of the low stimulus intensity with the high cue mean were

(qualitatively) at the level of the ratings of the high stimulus intensity with the low cue mean, in both modalities:

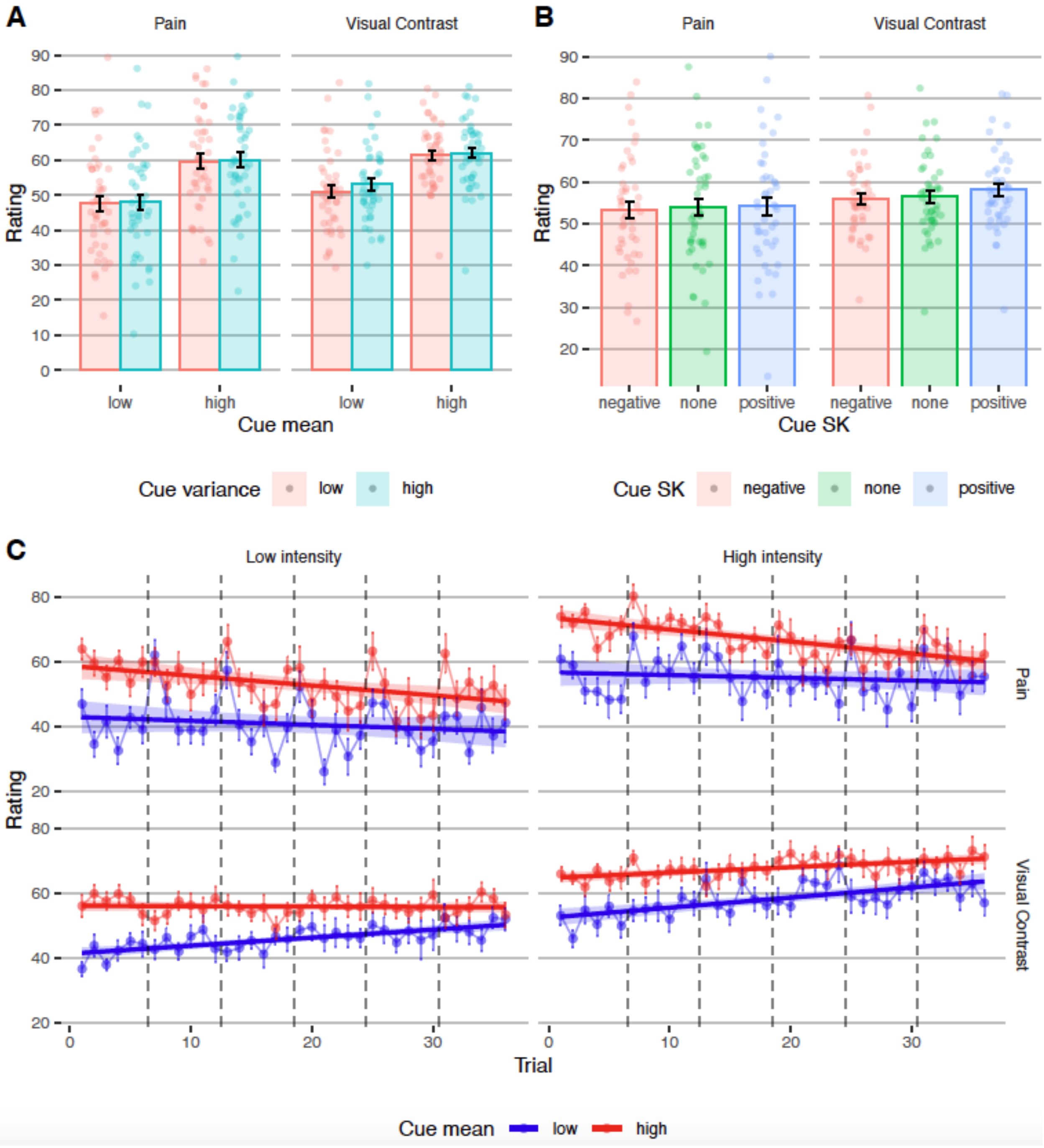

Figure 4. Behavioral results, cued-perception task. (**A**) Participants' perception ratings as a function of the mean (x axis) and variance (color) of the cue's values, in pain and visual trials (column). (**B**) Participants' perception ratings as a function of the skewness (x axis and color) of the cue's values, in pain and visual trials (columns). In panels A and B, bars represent averages across participants, error bars represent the standard error of the mean across participants, and points represent single participants. (**C**) The effect of cue mean (color) on pain and visual contrast ratings over time, for low vs.

high stimulus intensity (columns), in pain and visual perception (rows). Error bars represent standard error of the mean across participants. The lines represent the linear fit, and the shading represents 95% CIs of the linear fit. Vertical dashed lines separate between different blocks (note that a new skin site was used for each block, and thus the increase in the averaged pain rating for the first trial of each block stems from site-nonspecific sensitization and site-specific habituation [72]).

Comment 3.2

2. Please check your paragraph about effects of cue variance on expectations on page 6. You describe a significant cue mean x modality interaction. Do you mean cue variance x modality instead or a three-way-interaction? If the former, then one of the following sentences should also be saying that participants expected more intense stimuli after more certain cues, but that this was not true for visual stimuli. Additionally, the line of argumentation on why the cue mean and variance interaction should show up in the stimulus ratings but not in the expectations are a bit unclear, on page 11 you describe this much better.

Response 3.2

We thank the reviewer for pointing out this mistake in our original manuscript. As we noted in Response 1.6, we now focus on each modality independently and thus this result was removed from the text. Following your suggestion, we have now revised the description that was previously in page 6:

"***Effects of cue variance on expectations.*** *Previous studies have mostly focused on the effect of cue variance on perception rather than expectations. This is because the variance is thought to influence the precision (certainty) of the expectations, not their value. Therefore, cue variance should affect cue influences on perception, with more certain predictions (lower variance) having stronger effects.*" (page 8).

Comment 3.3

3. Please provide information on statistical thresholds used for the ROI analyses. Which significance level is used and how is the correction for multiple comparisons done?

Response 3.3

We present results both corrected and uncorrected for multiple comparisons in order to balance concerns related to Type I and Type II errors. It is standard practice to apply multiple comparison corrections when conducting multiple statistical tests to control for false positives (Type I error). However, it is important to note that our analyses involve a priori, planned tests in a limited number of predefined regions of interest. Many statisticians argue that correction for multiple comparisons is not strictly necessary in such cases, as long as all tests are planned in advance and fully reported [14,15]. For example, Gelman writes, "the original sin is selection. The problem with p-hacked work is not that p-values are uncorrected for multiple comparison, it's that some subset of comparisons is selected for further analysis"

(https://statmodeling.stat.columbia.edu/2017/06/25/analyze-comparisons-thats-better-looking-max-difference-trying-multiple-comparisons-correction/).

There are several reasons for considering such an approach. First, correcting across planned comparisons leads to inconsistencies across families of tests that can pose serious problems. Suppose we have a single finding that we have strong evidence for from previous studies and we perform a planned test for it in a new study. An effect size sufficient to achieve $p < 0.05$ is adequate. Now suppose we have 19 other findings, all of which we expect to replicate in the new study. With correction, our ability to confirm any one of them declines – even if they are all true, and even if they are independent. The more effects we know of, the less able we are to find anything. Second, correcting across a series of planned tests privileges control of Type I error at a severe cost in Type II error, particularly as the number of tests grows. We planned tests in these regions because we (a) expected moderate effect sizes in the brain based on large-sample reference studies, e.g., Cohen's $d = 0.5$ [16], and (b) committed to reporting effects of all tests (i.e., all regions) whether significant or not. The former implies a large increase in Type II error rates under multiple comparisons correction (e.g., 80% power for $N = 32$ for a planned test in a single region with $d = 0.5$, but 35% power when correcting across 20 tests). Null effects of predictive cues (mean and variance), as we mostly report here, are only meaningful if the test is sufficiently powered. This study was powered to test planned effects in a small set of regions at $p < 0.05$. And, put simply, the interesting observation here is that we observe no effects of cues in most of the brain regions we tested, a conclusion whose meaningfulness rests on having a sufficiently liberal threshold to achieve adequate power.

To find common ground between these schools of thought, we take a balanced approach by reporting both corrected and uncorrected results. This allows for transparency while ensuring that our statistical analyses remain interpretable within the context of our study design.

We revised the manuscript accordingly. For example:

Methods:

"*ROI analyses were conducted using a priori, planned comparisons in a limited number of predefined regions. Given that all tests were specified in advance and fully reported, multiple comparison correction was not strictly necessary [118,119]; however, to balance concerns related to Type I and Type II errors, we report both corrected and uncorrected results, by noting when a result is only significant with an uncorrected threshold (based on a Bonferonni correction within each set of regions). This approach ensures transparency while maintaining adequate statistical power to detect meaningful effects.*" (page 40)

Results:

"*Activity was higher for higher visual contrast in the right V1 (p = .041, only uncorrected, i.e., this result does not survive correction for multiple comparisons [see Methods]) and left V2 (p = .028, only uncorrected).*" (page 20)

"*Activity in all pain processing regions was again higher for higher noxious stimulus intensity (all ps ≤ .001), but only the PAG was sensitive to predictive cues: PAG activity was higher following low- vs. high-mean cues (p = .031, only uncorrected), consistent with aversive PE-like responses found in the PAG in previous studies [79,80]. In the visual perception task, activity in visual regions was only higher for higher visual contrast in V3 (right p = .046, left p = .017, both only uncorrected; all other ps ≥ .193).*" (page 20)

Discussion:

"*Which areas show brain responses that assimilate to predictions or contrast with them (encode PE) is currently an active area of investigation. A working hypothesis is that early sensory areas encode sensory PEs, as they are relatively distal from internal models and shielded from their effects, whereas higher-level perceptual areas encode posterior perceptions, which represent stimuli but assimilate to predicted values. This may be particularly true when internal predictive models are conceptual, including the kinds of non-reinforced cues about others' experience that we studied here. Here, PAG activity was higher following low vs. high cue mean, consistent with pain-related aversive PE that was found in the PAG in previous studies [79,80] (but this result did not survive correction for multiple comparisons). Together with the assimilation towards predicted values in higher-level systems, our results largely support this working hypothesis, although we do not directly test encoding of different types of PEs [90].*" (page 25)

Comment 3.4

4. In figure 2A, why were the five cue mean levels collapsed and which ones were collapsed? Could you also provide the full figure on this?

Response 3.4

We previously collapsed the two lower cue mean levels and the two higher cue mean levels to simplify visualization (the models included all levels). We have now revised the figure to show the 5 levels of cue mean included in our experiments:

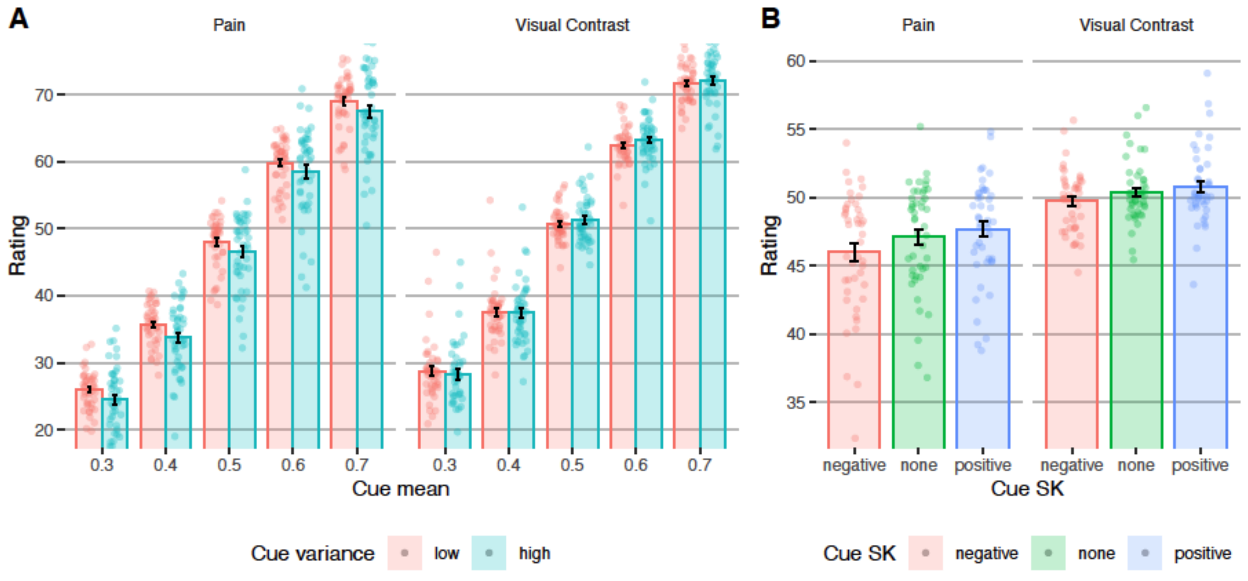

Figure 2. Behavioral results - expectation task. (**A**) Participants' expectation ratings as a function of the mean (five cue mean levels were collapsed into three for visualization) and variance of the cue's values, in pain and vision trials. (**B**) Participants' expectation ratings as a function of the skewness of the cue's values, in pain and vision trials. In both panels, bars represent averages across participants, error bars represent the standard error of the mean across participants, and points represent single participants.

Comment 3.5

5. Please also provide information on the blinding of the participant towards the paradigm structure. I assume they were not aware of the fact that there are only two or five intensities in each task involving stimuli? And did they believe that the presented cue distributions were real ratings? Because depending on this belief, they might integrate this information more or less strongly.

Response 3.5

We now mention in the Methods that participants were blinded to the design and manipulation. The cues were presented as ratings of previous participants and most participants believed they were, and they did not receive any information regarding the number of different intensities (or different cue means / variance / skewness).

Comment 3.6

6. Which thermode did you use for the heat stimulation? You mention the PATHWAY ATS but then describe very fast ramp-up times of 70°C/s, which to my knowledge are associated with the PATHWAY CHEPS model if I am not mistaken. Or do you have a newer model that can provide such timings?

Response 3.6

We thank the reviewer for pointing us to this error. We have used CHEPS thermodes, and have now corrected this information in the Methods section:

"*An fMRI compatible Peltier thermode (1.5 × 1.5 cm surface, PATHWAY CHEPS; Medoc, Inc, Israel) delivered heat to the left volar forearm of the participant in the stimulus-response task and the cued-perception task.*" (page 34)

Comment 3.7

7. Please refrain from comparing nociceptive and visual stimuli for example on page 13 when you discuss your supplementary results. As you correctly describe in the limitations section the scales of the stimuli are not comparable, and a stronger assimilation towards cued values does not necessarily mean that pain perception in general is more ambiguous but that the chosen pain stimuli used in this study were probably more ambiguous than the visual stimuli. When matching the ambiguity of the stimuli one could maybe see the same effects. The same can be said in response to your discussion of modality-specific vs modality-general effects on higher-level processing ROIs on page 23. Maybe the use of more ambiguous visual cues would lead to similar activations in higher-level processing regions as for the nociceptive stimuli.

Response 3.7

We now refrain from directly comparing the pain and visual modality (see response 1.2). We also revised the text to better contextualize the ambiguity of the noxious compared to the visual stimuli, and revised the discussion of modality-specific vs. modality-general effects on higher level processing ROIs:

Discussion

"*The persistent effects of the non-reinforced cues in both pain and visual perception might represent modulation of early perceptual processes, modulation of perceptual decision-making (higher-level affective and cognitive processes), or a post-experience report bias (i.e., demand characteristics [92], or conformity to alleged ratings of other participants [93]). Our neural results suggest that cues affect perceptual decision-making, since activity in regions related to early perceptual processing was not affected, while neural activity related to higher-level processing was affected by the cues, at least in pain. Moreover, neural effects were different between the two modalities. This could indicate either that expectation effects are modality-specific rather than modality-general, even when they operate on higher-level processes, or that there were other meaningful differences between the two modalities in our experiment, such as difference in ambiguity.*" (page 26)

Comment 3.8

8. Please clearly provide separate statements when you describe your expectation and your perception results, as it should be immediately clear to the reader that these experiments were done on a different day. For example, both in the abstract and in the discussion, you switch between both descriptions back and forth, so that one could be tempted to draw conclusions from one to the other. Yet I feel like there are different processes at work, and the discrepancy is interesting in itself. It would be interesting to hear also about your opinions regarding the discrepancy of expectation and perception effects. Are the effects of variance or skewness just to small to then have a notable effect when the expectation is weighted against the stimulus input? Or do participants learn over time to ignore the cues? Or do they use more elaborate models in a context where they are asked to rate the expectation but if the task demand is different, they use more simple heuristics to evaluate the percept? You tried to tackle some of these questions also in your supplementary analyses. I was expecting to see these in the main manuscript as the methods descriptions for that are also in the main manuscript, maybe move one of these. But then I also understand why they cannot capture all the questions one might have regarding the two processes of expectation and perception. One major issue for me in these models is the inability to capture the effect of cue variance on the integration of cue information as you also mention this already in the abstract as one of the main goals of the paper. This integration should be trial-specific yet the weightings of input and expectation are modelled the same for all conditions, or only decaying over time. It is also not so clear to me what can be learned in a paradigm in which the cues are non-informative. Nonetheless, it was great to see how different individuals' data fitted the models so differently which raises even more interesting questions.

Response 3.8

We thank the reviewer for these important comments. We now better explain the rationale for using unreinforced cues (see Response 1.1 and Response 1.8). We now also separate conclusions about expectations and perceptions more clearly, for example:

Abstract:

"*Forty-five participants observed cues–presented as ratings from 10 previous participants–with varying cue means, variances (precision), and skewness across trials. Participants reported expectations regarding the painfulness of thermal stimuli or the visual contrast of flickering checkerboards. In a second session, similar (sham) cues were each followed by a visual or noxious thermal stimulus. While perceptions assimilated to expected values in both modalities, this effect was stronger for more precise cues only in visual perception, while participants reported slightly higher pain following more uncertain cues... Furthermore, behavioral and computational models of the expectation session revealed that expectations were biased towards extreme values in both modalities, and towards low-pain cues specifically.*"

Discussion:

"*Consistent with these findings, in the current study, reported perceptions showed strong assimilation towards predicted values (cue mean) in both pain and visual intensity judgments.… Finally, we found that when generating expectations, people are strongly drawn towards the cue mean, but overweight extreme values across modalities, and also smaller values specifically in pain.*" (page 23)

We now also elaborate on the differences between the effects of the cues on expectation generation vs. perception in the discussion:

Discussion:

"*There are several potential explanations for the discrepancy between cue effects on expectation ratings and perceptual judgments. Participants may rely on different cognitive strategies when explicitly rating their expectations versus when making perceptual judgments. When explicitly asked to report expectations, participants might engage in a more deliberate, model-based process that incorporates cue information differently than when making perceptual decisions, which could rely more on heuristics or sensory evidence. Prior research suggests that the act of making expectancy ratings itself can alter learning and decision-making processes, potentially by encouraging a more reflective or abstract representation of the cue-stimulus relationship [98]. This could mean that expectation ratings reflect an explicit cognitive model of the task, whereas perceptual judgments are more influenced by direct sensory experience and task demands. Another possibility is that the effects of variance or outliers in the cue distributions may be too subtle to influence perceptual judgments once expectation is weighted against stimulus input. The predictive influence of expectation might be diminished when it does not provide strong enough information to override perceptual uncertainty. Alternatively, participants may learn over time to downweight or ignore cues if they prove to be unreliable predictors of the actual stimulus, leading to reduced cue effects on perception even if they still influence explicit expectation ratings; however, our results indicate that the effect of the cue persisted, and thus this explanation is less likely. Further work is needed to disentangle whether this discrepancy arises from differences in cognitive strategies, cue weighting, or the extent to which expectations interact with sensory processing in a given task context.*" (page 28)

Comment 3.9

9. Could you provide data on the scaling factors described on page 32? It would be interesting to see how the different day and context changes the ratings especially for pain as I would expect some stronger fluctuations within an individual than in vision.

Response 3.9

In each of the five computational models we have tested for the perception data, there is a scaling factor per participant and modality. Thus, the statistics of the scaling factor depend on the model of choice. To report some relevant statistics, here we chose the best fitting model per individual, and used the scaling factor from this model:

Supplementary Information:
"*Based on each participant's scaling factors from their own best fitting model, ratings were higher in the perception session (performed in the MRI scanner) compared to the first session in both modalities (both scaling factors larger than 1; pain: M = 1.42, SD = 0.59, t(44) = 4.747, p < .001; visual perception: M = 1.44, SD = 0.43, t(44) = 6.921, p < .001), and the scaling factor did not differ significantly between pain and visual perception (t(44) = -0.24, p = .809).*" (Supplementary Information, page 5)

Comment 3.10

10. Please make it clearer that the brain imaging results are based on the regressor for the stimulation. This only becomes evident after reading that you also modelled the cue period in the supplement data.

Response 3.10

Thank you, we now clarify this up front:

Results:
"*We computed a dot product based score, the 'pattern response', for the stimulus period of each trial…*" (page 17)
"*To complement neuromarker analyses, which capture activity in distributed systems, we tested whether the cues affected neural responses to the stimuli in several a priori ROIs related to pain and visual perception (Figure 6; for all regions see Table S2).*" (page 20)

Methods:
"*The first level (run level) GLM model included two regressors per trial (48 regressors of interest per run in total): separate regressors for each cue period and for each stimulation period (single trial model, or a "Beta series" [117])...*" (page 39)

Comment 3.11

11. Did the participants experience the cues already with real stimuli when they were practicing the cued-perception task outside the scanner? Can you describe how long they practiced?

Response 3.11

Yes, the participants experienced the cues with real stimuli during practice, which included six trials overall (three from each modality). We have now added this information to the Methods section:

"...followed by a brief practice of the cued-perception task outside the scanner (including the cues and experiencing actual stimuli), which included six trials overall (three from each modality)." (page 30).

Comment 3.12

12. The last sentence in the abstract seems a bit strong/general to me, as you mention the effects of expectations would operate on higher-level processes, yet you mentioned in your discussion that this could also completely differ when using a different type of expectations. Especially, when using non-informative, non-reinforced cues I would expect cues to have less of an impact on lower-level processes, but would not completely dismiss the idea of possible effects there. You also mention in this sentence that selective attention would provide a better fit to your data, yet this is not mentioned in your manuscript. Can you elaborate on that?

Response 3.12

We agree with the reviewer, and have tuned down the conclusion regarding the higher-level processes and clarified what we meant by "selective attention":

Abstract:
"*Our findings suggest that predictive processing theories should be combined with mechanisms such as selective attention (e.g., to outliers) to better fit empirical findings, and that expectation generation and its perceptual effects are mostly modality-specific and operate on higher-level processes rather than early perception, at least when cues are not reinforced.*".

When discussing the findings regarding lower vs. higher-level neural processing, we explain that these results might depend the cues used:

Discussion:
"*Here, early nociceptive and visual regions – and a neuromarker for nociceptive pain, the NPS – did not show evidence for assimilation towards predicted values during perception, nor did higher-level perceptual regions such as the aMCC and aIns in pain or V3, V4 and V5 in visual perception. Only the right NAc and SIIPS, a distributed neuromarker for pain related to endogenous sources (including signal in dmPFC, aIns, vmPFC, and other regions), showed significant (bot relatively weak) assimilation to predicted values. These*

results suggest that early perceptual processes are relatively shielded from influences of conceptually driven predictions, and the strong effects on perception are driven by higher-level evaluative processes, in line with psychological accounts questioning early influences of context information on perception [59] and recent findings in the context of placebo analgesia [63]. Such effects might also depend on the type of cues used. Indeed, a previous study using cues similar to those we used here has found that informational cue effects were mediated by frontoparietal areas involved in high-level construction of perception and value (e.g., dlPFC, OFC and IPS), whereas classically conditioned cue effects were mediated by subcortical systems [10]." (page 25)

Comment 3.13

13. I was a bit puzzled about the results of the computational model of expectations in figure 3B. To me it seems that the model for visual stimuli predicts somewhat distinct clusters of responses at the position of the cue means. Why is that? Is it something in the participant's rating behavior that is different for the visual expectation task, maybe because they use some knowledge of the previous stimulus intensity task? I would expect comparable results for both modalities on naïve participants, yet their previous experience of the stimuli and scale use has changed their behavior in the expectation task. Even the model performance is better in pain data. Why is that?

Response 3.13

There are indeed distinct clusters based on the cue mean values, in both modalities but more so in the visual modality. As explained in Response 2.6, the correlation between the observed and predicted values is still significant for both modalities within each cue mean level. We believe that this clustering is more substantial in the visual modality because participants are more affected by other properties of the cue (e.g., outliers or smaller values) in pain vs. vision. This might stem from participants viewing themselves as less sensitive to pain compared to others or from participants looking for "safety signals" specifically in pain.

Comment 3.14

14. Why did you not detect a prediction error response in the anterior insula as in previous investigations (e.g., Geuter et al., 2018, eLife; Fazeli & Büchel 2018; Journal of Neuroscience)? Is it mainly because the cue vs stimuli relation is much different in these paradigms and participants in these studies do not integrate the cue information into their percept but are mainly showing a strong mismatch between the cue information and the stimulus input?

Response 3.14

As the reviewer correctly notes, we have much to learn about where prediction errors are generated in the brain with different types of cues and contexts. Depending on the level of influence of a cue, PEs could arise or not. If an area like insula receives input from the cue and stimulus, a PE is likely. But if cues only affect downstream/higher-level areas (e.g., prefrontal cortex), a PE may not be generated in the insula. A full theory of what the neural levels might be and which types of cues affect which levels has not yet been developed, but is an important future direction (see also Response 1.15).

Comment 3.15

15. In figure 4C you nicely describe that the first trial of each run shows an increased average pain rating because a new skin site is used. As you also exclude two training trials in the previous stimulation task, it might be reasonable to also exclude the first trials here? Have you tried this? I feel like it as a huge influence on the results and might increase the noise in your data when it is not being modelled.

Response 3.15

As the reviewer correctly notes, trial (within skin site) is an important variable, particularly in pain perception. As explained in detail in Response 1.3 and Response 1.13, we now control for block and trial effects in our models, and have revised our methods, results and conclusions accordingly.

Comment 3.16

16. Just out of curiosity and because we sometimes acquire similar data: do you have the skin conductance results available? Are they also shifted towards the cue mean or only showing effect of stimulus intensity?

Response 3.16

We did not analyze the skin conductance data, and they are beyond the scope of the current study. We hope to have the resources to do further analyses of skin conductance and computational models in the future. However, we have found in previous studies using similar unreinforced cues that autonomic responses shift towards the cue mean in pain [7] and vicarious pain [17]. These previous findings also underscore that even the unreinforced cues we use here exert strong and meaningful effects on participants' responses.

References

1. Hird EJ, Charalambous C, El-Deredy W, Jones AKP, Talmi D. Boundary effects of expectation in human pain perception. Sci Rep. 2019;9: 9443.

2. Yoshida W, Seymour B, Koltzenburg M, Dolan RJ. Uncertainty increases pain: Evidence for a novel mechanism of pain modulation involving the periaqueductal gray. J Neurosci. 2013;33: 5638–5646.

3. Roy M, Shohamy D, Daw N, Jepma M, Wimmer GE, Wager TD. Representation of aversive prediction errors in the human periaqueductal gray. Nat Neurosci. 2014;17: 1607–1612.

4. Jepma M, Koban L, van Doorn J, Jones M, Wager TD. Behavioural and neural evidence for self-reinforcing expectancy effects on pain. Nature Human Behaviour. 2018;2: 838–855.

5. Pavy F, Zaman J, Von Leupoldt A, Torta DM. Expectations underlie the effects of unpredictable pain: a behavioral and electroencephalogram study. Pain. 2024;165: 596–607.

6. Grau JW, Hudson KE, Johnston DT, Partipilo SR. Updating perspectives on spinal cord function: motor coordination, timing, relational processing, and memory below the brain. Front Syst Neurosci. 2024;18: 1184597.

7. Koban L, Wager TD. Beyond conformity: Social influences on pain reports and physiology. Emotion. 2016;16: 24–32.

8. Rao RP, Ballard DH. Predictive coding in the visual cortex: a functional interpretation of some extra-classical receptive-field effects. Nat Neurosci. 1999;2: 79–87.

9. Summerfield C, de Lange FP. Expectation in perceptual decision making: neural and computational mechanisms. Nat Rev Neurosci. 2014;15: 745–756.

10. Heilbron M, Chait M. Great expectations: Is there evidence for predictive coding in auditory cortex? Neuroscience. 2018;389: 54–73.

11. Denham SL, Winkler I. Predictive coding in auditory perception: challenges and unresolved questions. Eur J Neurosci. 2020;51: 1151–1160.

12. Spitzer B, Waschke L, Summerfield C. Selective overweighting of larger magnitudes during noisy numerical comparison. Nat Hum Behav. 2017;1: 145.

13. Jepma M, Jones M, Wager TD. The dynamics of pain: evidence for simultaneous site-specific habituation and site-nonspecific sensitization in thermal pain. J Pain. 2014;15: 734–746.

14. Keppel G. Design and analysis. 3rd ed. Upper Saddle River, NJ: Pearson; 1991.

15. Rosenthal R, Rosnow RL. Contrast analysis. Cambridge, England: Cambridge University Press; 1985.

16. Poldrack RA, Baker CI, Durnez J, Gorgolewski KJ, Matthews PM, Munafò MR, et al. Scanning the horizon: Towards transparent and reproducible neuroimaging research. Nat

Rev Neurosci. 2017;18: 115–126.

17. Zhang L, Wager TD, Koban L. Social cues influence perception of others' pain. Eur J Pain. 2024;28: 845–854.
